# Psychometric properties of the German version of the brief resilience scale in persons with mental disorders

**DOI:** 10.1186/s12888-024-06062-x

**Published:** 2024-09-27

**Authors:** Jan Broll, Sarah K. Schäfer, Andrea Chmitorz, Adrian Meule, Ulrich Voderholzer, Isabella Helmreich, Klaus Lieb

**Affiliations:** 1 Leibniz Institute for Resilience Research (LIR), D–55122 Mainz, Germany; 2 Clinical Psychology, Psychotherapy and Psychodiagnostics, Technical University of Braunschweig, D–38106 Braunschweig, Germany; 3 Faculty of Social Work, Education and Nursing, Esslingen University of Applied Sciences, D–73728 Esslingen, Germany; 4 Department of Psychology, University of Regensburg, D–93053 Regensburg, Germany; 5 Schoen Clinic Roseneck, D–83209 Prien, Germany; 6 Department of Psychiatry and Psychotherapy, University Hospital of the LMU Munich, D–80336 Munich, Germany; 7 Department of Psychiatry and Psychotherapy, Faculty of Medicine, Medical Center, University of Freiburg, D–79085 Freiburg, Germany; 8 Department of Psychiatry and Psychotherapy, University Medical Center of the Johannes Gutenberg University Mainz, D–55131 Mainz, Germany

**Keywords:** Resilience, Brief resilience scale, Psychometrics, Assessment, Mental health

## Abstract

The Brief Resilience Scale (BRS) was developed to assess individual differences in the ability to recover from stress despite adversity and has been translated into several languages. This study aimed to examine the psychometric properties (i.e., item characteristics, reliability, factor structure, measurement invariance, and validity) of the German version of the BRS in persons with mental disorders. A total of *N* = 5,986 persons admitted to inpatient treatment completed the German version of the BRS and other questionnaires. The discriminating power of the items, the difficulty of the items, and the internal consistency were all sufficient. Moreover, confirmatory factor analysis supported the two–factor structure of the BRS, consistent with the findings of the German validation study in a non–clinical sample. The BRS also had strict measurement invariance across diagnostic groups for mental disorders according to ICD–10. Validity was examined using a network analysis, in which the BRS demonstrated positive correlations with life satisfaction, self–efficacy and optimism and negative correlations with somatic symptoms, anxiety, insomnia, and depression. The BRS can serve as a reliable and valid tool for assessing resilience in clinical settings, facilitating the identification of persons with potentially lower psychosocial resources.

## Introduction

Psychological resilience has gained significant attention in the field of psychology due to its important role in promoting mental wellbeing and positive psychological outcomes [1–3]. The term resilience as an outcome refers to good mental health despite stress, that is, the maintenance or quick recovery of mental health during or after exposure to major stressors or chronically heightened levels of daily hassles [2, for a critical discussion see: 4, 5]. Resilience was traditionally perceived as a stable trait inherent in individuals, however, recent research views resilience as an adaptive outcome that can be developed and nurtured through various experiences and interventions [2, 4, 5]. Over the years, various questionnaires were developed to assess resilience [6–8]. Most of them are based on a trait–oriented approach — for example, the Resilience Scale by Wagnild and Young [9] – or assess the availability of protective factors and resources to maintain or regain mental health despite adversity like the Connor–Davidson Resilience Scale [10]. To date, there is no gold standard to measure resilience [6–8]. Smith and colleagues [11] developed the Brief Resilience Scale (BRS). It consists of six items that are rated on a five–point scale and measures the ability to bounce back from stress. According to Smith et al. [11], measuring the ability to recover or “bounce back” from stress emphasizes an outcome–oriented interpretation of resilience.

The BRS has been translated into several languages including Arabic [12], Chinese [13], Czech [14], Dutch [15], Korean [16], Malaysian [17], Polish [18], Slovak [14], Spanish [19], and German [20]. The internal consistency was heterogenous across studies (*α* = 0.56–0.93) and it showed acceptable retest reliability (intraclass correlation coefficient [ICC] = 0.69–0.94). Factor analyses showed that the BRS is a unidimensional scale, with scores being associated with other resilience measures, (mental) health outcomes or social support [11].

The psychometric properties of the German version of the BRS [20] were examined in a population–based sample (*N* = 1,481). A two–factor model yielded superior model fit with a factor for general resilience (items 1–6) and a method factor controlling for method effects due to item wording (items 2, 4, 6). Internal reliability was *ω* = 0.85. The BRS was positively correlated with wellbeing, social support, and optimism and negatively correlated with somatic symptoms, anxiety, insomnia, social dysfunction, and depression. In another study, Kunzler et al. [21] analyzed the German version of the BRS in greater detail with regard to construct validity. Based on latent and manifest correlations, the convergent and discriminant validity of the BRS were fair to good: it showed negative correlations with perceived stress and external locus of control, and positive correlations with optimism, self–efficacy, and internal control beliefs. Previous studies supported the psychometric properties (e.g. reliability, factorial validity, convergent validity) of the BRS in different samples such as persons with HIV infection, cancer patients, parents of children with disabilities [19] and persons with mental disorder [22].

The current study focused on people with mental disorders who were undergoing psychotherapeutic treatment. Validating the BRS in such a sample holds significant clinical implications. Individuals seeking mental health services often face unique challenges. By testing the psychometric properties of the BRS in this population, mental health practitioners can assess levels of resilience, identify individuals with lower psychosocial resources and longitudinally monitor alterations in resilience over time. Resilience is an important construct in the field of mental health and may be useful for the treatment of mental disorders. Reviews have demonstrated a general efficacy of interventions to promote resilience [23, 24]. Previous literature indicates that psychological resilience acts as a buffer between stress and mental health outcomes [25, 26], potentially mitigating the negative effects of stress [27, 28]. Both cross–sectional and longitudinal studies provide evidence for resilience mediating the influence of personality traits, e.g. neuroticism, and harmful family dynamics on depressive symptoms and sleep quality [29, 30], and reducing the risk of depression in individuals with adverse childhood experiences [27, 31]. A lack of resilience during adolescence was found to be associated with a higher likelihood of prolonged use of antidepressant and anxiolytic medications in clinical populations [32]. A recent study of people with mental disorders showed that higher resilience at the start of treatment was associated with a greater decrease in depressive symptoms, while higher depressive symptoms predicted a smaller increase in resilience, highlighting the dynamic interplay between resilience and depression during treatment [33].

## Aims und objective

First, we analyzed the item characteristics (item difficulties, item discrimination) of the German BRS in a sample of persons with mental disorders. Second, we examined the internal consistency of the BRS. Third, we focused on investigating the factorial validity of the BRS using confirmatory factor analysis. Fourth, we conducted an analysis of measurement invariance across diagnostic groups based on the International Classification of Diseases (ICD-10; [34]) criteria. Fifth, we examined the convergent validity by investigating the associations between the BRS and related constructs, such as mental health, coping strategies, social support, and optimism, using a network modeling approach.

## Method

### Participants

The present study entails the examination of questionnaires completed by 5,986 persons with mental disorders in a psychiatric clinic situated in Southern Germany. Data collection occurred at admission and discharge, spanning from February 2020 to February 2023. We used only respondents’ admissions data for our analyses.

### Study design

At the clinic, sociodemographic and diagnostic data such as age, sex, diagnoses, medication, length of stay, and questionnaire scores are transferred to a database. This database allows for the export of data without personally identifiable information, ensuring the privacy and confidentiality of patients. Moreover, in accordance with the guidelines of the ethics committee at the Ludwig Maximilians University, Munich, retrospective studies conducted using pre–existing, anonymized data are exempt from ethics approval.

### Materials

**Demographic Data**. Information about age, sex, and mental disorder diagnoses according to ICD–10 [33] were obtained from the clinical records of the hospital.

**Brief Resilience Scale**. The perceived ability to recover from stress was assessed using the German translation of the BRS [20]. Six items are rated on a five–point scale (1 = strongly disagree; 5 = strongly agree). Items 2, 4, and 6 are negatively phrased, with higher scores indicating lower levels of resilience. These items were recoded to calculate the mean (range: 1–5). Higher total scores indicate a higher ability to recover from stress.

**Patient Health Questionnaire–depressive symptom severity scale (PHQ–9).** The PHQ–9 [35] is a widely used self–report screening tool designed to assess the severity of depressive symptoms. Respondents are asked to rate the frequency of each symptom over the past two weeks on a scale from not at all (0) to nearly every day (3). The total sum score can range from 0 to 27, with higher scores indicating more severe depressive symptoms. The internal consistency in our sample was found to be acceptable (*ω* = 0.85, 95% CI [0.84; 0.85]).

**Generalized Anxiety Disorder Scale–7 (GAD–7)**. The GAD–7 [36] is a questionnaire designed to assess the severity of anxiety symptoms. It consists of seven items presented in the format of statements describing symptoms associated with anxiety disorders. Respondents are asked to rate the frequency of each symptom over the past two weeks on a scale from not at all (0) to nearly every day (3). The sum score ranges from 0 to 21, with higher scores indicating more severe anxiety symptoms. In our sample, the GAD–7 demonstrated acceptable internal consistency (*ω* = 0.85, 95% CI [0.84; 0.85]).

**Patient Health Questionnaire–somatic symptoms severity scale (PHQ–15)**. The PHQ–15 [37] is a questionnaire that assesses somatic symptoms associated with various medical conditions. It consists of 15 items presented in the format of physical symptoms commonly experienced by individuals. Respondents are asked to rate the extent to which they have been bothered by each symptom over the past four weeks on a scale ranging from not bothered at all (0) to bothered a lot (2). The total score can range from 0 to 30, with higher scores indicating greater severity of somatic symptoms. The internal consistency within our sample was determined to be acceptable (*ω* = 0.80, 95% CI [0.80; 0.82]).

**Insomnia Severity Index (ISI)**. The ISI [38] assesses the severity of insomnia symptoms and the impact of insomnia on individuals’ daily functioning. It consists of seven items that capture various aspects of insomnia on a scale from none (0) to very severe (4). The total score ranges from 0 to 28, with higher scores indicating a higher level of insomnia symptoms. We observed acceptable internal consistency in our sample (*ω* = 0.89, 95% CI [0.88; 0.90]).

**Satisfaction with Life Scale (SWLS)**. Patients overall satisfaction with their life was measured using the SWLS [39]. Five items are rated on 7–point scale from strongly disagree (1) to strongly agree (7). The total score ranged from 5 to 35, with higher scores indicating higher levels of life satisfaction. The internal consistency of our sample was found to be good (*ω* = 0.86, 95% CI [0.85; 0.86]).

**Optimism–Pessimism Scale–2 (SOP–2)**. Patients’ dispositional optimism and pessimism was measured using the SOP–2 [40]. The two items are rated on a 7–point scale. Higher scores indicate more dispositional optimism. Internal consistency was acceptable in our sample (*ω* = 0.84, 95% CI [0.83; 0.86]).

**General Self–Efficacy Short Scale–3 (GSE–3)**. Patients’ perceived self–efficacy beliefs were measured using the GSE–3 [41]. Three items are rated on a five–point scale ranging from do not agree at all (1) to completely agree (5). Higher scores indicate stronger self–efficacy beliefs. In our sample, the internal consistency of the measure was acceptable (*ω* = 0.84, 95% CI [0.83; 0.85]).

### Data analyses

Analyses were conducted using R version 4.2.1 [42] and the packages *easystats* [43], *tdiyverse* [44], *psych* [45] *lavaan* [46], *semPlot* [47], *bootnet* [47], *qgraph* [48], and *sjPlot* [49].

To evaluate the item characteristics of the BRS, we calculated mean (M) and standard deviation (SD) of each BRS item, which provided information on the average response and the extent of variability within the sample. Additionally, we examined the skewness of the item distributions to assess the distribution of responses per item. Furthermore, we computed item difficulty, which refers to proportion or percentage of respondents who answered a specific item in terms of a higher level of the measured characteristic. We calculated the mean (M) and standard deviation (SD) for each item across each diagnosis group, providing insight into the average responses and the variability within the sample. Lastly, we calculated item discrimination, which measured the extent to which each item can differentiate between individuals with high and low levels of the measured trait.

To assess the internal consistency of the BRS, we employed two commonly used measures of internal consistency: Cronbach’s alpha α [50] and McDonald’s omega ω [51]. Cronbach’s alpha considers each item of a scale as an independent part. The prerequisite is the strict, and often not fulfilled, assumption that the covariances between all items are identical. In contrast, McDonald’s omega imposes no equality restrictions on the item parameters [52].

The factor structure of the BRS was tested with confirmatory factor analyses with maximum likelihood estimations, covariance matrices, and the Satorra–Bentler method of estimation to account for potential non–normality in the distribution of the data. Given the factor structure identified in previous research [20], we fitted two models: (1) a one–factor–model of general resilience; (2) a two–factor model with general resilience (item 1, item 2, item 3, item 4, item 5, item 6) and a method factor (item 2, item 4, item 6) reflecting the positive and negative wording of the items. To evaluate and compare the fit of different models, we used several commonly used goodness–of–fit indices and standard cut–off criteria [53]: the χ^2^ statistics, the Root Mean Square Error of Approximation (RMSEA; good fit < 0.06), the Comparative Fit Index (CFI; good fit: > 0.95), and the Standardized Root Mean Squared Residual (SRMR; good fit < 0.08). As the likelihood ratio (LR) test is overly sensitive in large samples [54, 55], we opted for the changes in the Comparative Fit Index (CFI) to assess model comparisons [54, 56].

Measurement invariance refers to the property of a psychological scale that ensures its validity remains consistent across different groups or conditions, allowing meaningful comparisons and interpretations of the data [57]. When analyzing measurement invariance, individuals in the ‘Other’ diagnosis group were excluded due to the lack of meaningful content interpretation for this category and the small number of cases. Similarly, the group ‘Disorders of adult personality and behaviour’ (F6) was excluded because the small number of cases would limit the reliability of the results. A series of confirmatory factor analyses were conducted to examine measurement invariance across the remaining different diagnostic groups. First, we assessed configural invariance, which tests whether the factor structure is equivalent across groups. Second, we examined metric measurement invariance, which tests whether factor loadings are equal between groups. Third, we assessed scalar invariance, which tests whether the intercepts of the items are equal across groups. Fourth, we examined strict invariance, which tests whether residual variances of the items are equivalent across groups. To be able to compare the different model with each other, we calculated χ^2^ statistics, RMSEA, CFI, and SRMR, with the cut–offs defined above [53]. Again, we compared models based on changes in CFI.

To examine the convergent and discriminant validity of the BRS, we employed a network analysis [58]. A network is a system comprising nodes (circles) connected with edges (lines) denoting the strength of connections between nodes. In psychological network models, nodes correspond to observed variables, and edges are used to represent the strength of associations between two variables [59]. In our network model, we incorporated several established measures of mental health and resilience, including symptoms of depression, anxiety, and insomnia, as well as somatic symptoms, life satisfaction, optimism, and self–efficacy. To capture the underlying associations among these variables, we utilized partial correlations, which account for the shared variance between variables while controlling for the influence of other variables in the model. Node placement was achieved using the Fruchterman and Reingold [60] algorithm. We used the EBICglasso (Extended Bayesian Information Criterion with the graphical lasso) method for model estimation. The EBICglasso combines the graphical lasso, a regularization technique for estimating precision matrices, with the Extended Bayesian Information Criterion, a model selection criterion [47]. The resulting network exhibits edges between nodes, which signify conditional independence relationships among the nodes, specifically representing partial correlations between pairs of nodes while accounting for the influence of all other nodes in the network. The network shows green and red connections between nodes that are indicative of positive and negative relations, respectively. Stronger relationships are shown in terms of thicker lines and denser colors, and nodes with stronger similarities are placed closer together.

## Results

### Sample characteristics

Table 1 presents the sample characteristics and diagnosis categories according to ICD–10 [34]. Most of the participants were female (76.95%), with males accounting for 23.05% of the sample. The average age was 32.13 years (SD = 16.59), and the age distribution ranged from 12 to 88 years, with a median age of 25. Diagnostic classifications according to ICD–10 revealed that affective disorders (F3) were the most prevalent (41.73%), followed by neurotic, stress–related, and somatoform disorders (F4, 24.91%) and behavioral syndromes associated with physiological disturbances and physical factors (F5, 32.23%). Disorders of adult personality and behavior (F6) constituted a smaller proportion (0.95%), while a minimal percentage fell into the “Others” category (0.18%). The mean treatment duration was 71.45 days (SD = 43.84), with a median of 64 days (range: 1 to 366 days).

**Table 1:** Descriptive statistics of the sample

	Overall (*N* = 5,986)
**Sex**	
Female	4,606 (76.95%)
Male	1,380 (23.05%)
**Age**	
Mean (*SD*)	32.13 (16.59)
Median (*Min; Max*)	25 (12; 88)
**Diagnosis according to ICD–10 (frequency**, **%)**	
Affective disorders (F3)	2,498 (41.73%)
Neurotic, stress–related and somatoform disorders (F4)	1,491 (24.91%)
Behavioral syndromes associated with physiological disturbances and physical factors (F5)	1,929 (32.23%)
Disorders of adult personality and behavior (F6)	57 (0.95%)
Others	11 (0.18%)
**Treatment duration (days)**	
Mean (*SD*)	71.45 (43.84)
Median (*Min; Max*)	64 (1; 366)

*Notes. SD*, Standard Deviation; *Min*, minimum; *Max*, maximum

### Item characteristics

Table 2 presents the item characteristics of the BRS at admission. The item difficulties for the BRS items ranged from 0.46 to 0.49 and were in the medium range. Items with a difficulty level of approximately 0.50 are favored in classical test theory due to their higher information content [61]. The item discrimination values for the BRS items ranged from 0.43 to 0.60. Values above 0.30 are considered to be good in classical test theory [61]. That suggest that the BRS items exhibited a moderate to high level of discrimination power. The calculated skewness values ranged from 0.53 to 0.61. These positive skewness values indicate that the item response distributions were slightly skewed towards the right side (i.e., respondents had a general tendency to endorse the statements).

**Table 2:** Item characteristics of the BRS items

Item	Mean (SD)	Skewness	Item Difficulty	Item Discrimination
1. I tend to bounce back quickly after hard times.	2.40 (1.05)	0.53	0.48	0.58
2. I have a hard time making it through stressful events.*	2.36 (*1.21*)	0.61	0.47	0.43
3. It does not take me long to recover from a stressful event.	2.42 (*1.12*)	0.60	0.48	0.45
4. It is hard for me to snap back when something bad happens.*	2.43 (*1.15*)	0.53	0.49	0.51
5. I usually come through difficult times with little trouble.	2.39 (*1.06*)	0.57	0.48	0.56
6. I tend to take a long time to get over set–backs in my life. *	2.28 (*1.06*)	0.61	0.46	0.60

*Notes*. * = reverse–coded

In Table 3, the means and standard deviations for each item of the Brief Resilience Scale (BRS) are reported across different diagnostic groups.

**Table 3:** Means and Standard Deviations of BRS items by Diagnostic Group

Diagnostic Group	BRS Item 1	BRS Item 2*	BRS Item 3	BRS Item 4*	BRS Item 5	BRS Item 6*
Affective disorders (F3)	2.26 (1.02)	2.31 (1.23)	2.31 (1.12)	2.37 (1.14)	2.37 (1.07)	2.19 (1.04)
Neurotic, stress-related and somatoform disorders (F4)	2.34 (1.03)	2.31 (1.23)	2.32 (1.12)	2.33 (1.19)	2.31 (1.06)	2.22 (1.06)
Behavioral syndromes associated with physiological disturbances (F5)	2.63 (1.05)	2.47 (1.17)	2.63 (1.09)	2.58 (1.11)	2.49 (1.05)	2.44 (1.08)
Disorders of adult personality and behavior (F6)	2.23 (1.12)	2.35 (1.36)	2.26 (1.08)	2.09 (1.07)	2.09 (1.04)	2.00 (1.04)
Others	2.55 (1.37)	3.00 (1.73)	2.82 (1.33)	2.89 (1.27)	2.80 (1.48)	2.70 (1.25)

*Notes*. * = reverse–coded

BRS scores varied between the different diagnostic groups. For people with affective disorders (F3), mean BRS scores ranged from 2.19 to 2.37, with standard deviations between 1.02 and 1.14. People with neurotic, stress-related and somatoform disorders (F4) had similar mean scores, ranging from 2.22 to 2.34, with standard deviations of 1.03 to 1.23. In contrast, those with behavioral syndromes associated with physiological disturbances (F5) had mean scores ranging from 2.44 to 2.63 and standard deviations between 1.05 and 1.17. For disorders of adult personality and behavior (F6), mean scores ranged from 2.00 to 2.35, with higher variability as indicated by standard deviations ranging from 1.04 to 1.36.

### Reliability

Internal consistency was good using Cronbach’s *α*, with *α* = 0.79, 95% CI [0.77; 0.80]. The McDonald’s omega coefficient was identical, *ω* = 0.79, 95% CI [0.76; 0.80], further supporting the internal consistency of the scale.

### Factor structure

As indicated in Table 4, the method–factor model (model 2) fitted the data significantly better than the one–factor model (model 1). The difference in the CFIs between the two models was 0.03, indicating a significant improvement in model fit when employing the bifactorial structure with method and general resilience factors.

**Table 4:** Results from confirmatory factor analyses and model comparisons

Model	χ^2^	df	*p*	RMSEA	CFI	SRMR	BIC	∆CFI
1) One Factor	405.35	9	< 0.001	0.087	0.951	0.037	98329.714	
2) Two Factors (method factor)	154.58	6	< 0.001	0.065	0.982	0.022	98104.93	0.03

*Notes*. χ^2^ = Chi squared; *df* = degrees of freedom; *RMSEA* = Root Mean Square Error of Approximation; *CFI* = Comparative Fit Index; *SRMR* = Standardized Root Mean Squared Residual; *BIC =* Bayesian Information Criterion; RMSEA and χ^2^ indicate Satorra–Bentler–scaled values; *∆CFI* = Difference between Comparative Fit Indices; Model 1 = one–factor model of general resilience (items 1–6); Model 2 = two–factor model of general resilience (items 1–6) and a method factor reflecting the positively and negatively worded items

We examined the factor loadings of the observed variables on the latent factors. The factor loadings represent the strength and direction of the relationships between each item and the underlying factors. The results, presented in Table 5, indicated that the loadings were satisfactory. All loadings consistently exceeded a magnitude of 0.50, demonstrating a strong association between the items and the respective underlying construct.

**Table 5:** Factor loadings of the BRS items

Item	Factor 1 (method factor)	Factor 2 (general resilience factor)
I tend to bounce back quickly after hard times	0.00	0.71
I have a hard time making it through stressful events*	0.18	0.52
It does not take me long to recover from a stressful event	0.00	0.55
It is hard for me to snap back when something bad happens*	0.79	0.51
I usually come through difficult times with little trouble	0.00	0.68
I tend to take a long time to get over set–backs in my life*	0.17	0.67

*Notes*. * = reverse–coded

### Measurement invariance

The results of the measurement invariance analyses are presented in Table 6. The CFI meet the recommended threshold of 0.95, indicating good model fit, the RMSEA values in the metric, scalar, and strict models were all below the desirable threshold of 0.080, suggesting good fit. The SRMR was consistently below the recommended 0.080 in all models, further supporting the overall good fit. The change in CFI was consistently small, with differences below 0.010 for each model comparison. As a result, we opted in favor of the model with the most constraints, the strict measurement invariance model.

**Table 6:** Measurement invariance analysis results

Model	χ2	df	*P*	RMSEA	CFI	SRMR	BIC	∆CFI	Accepted?
configural	170.34	18	< 0.001	0.067	0.98	0.020	96967.16		yes
metric	204.80	32	< 0.001	0.053	0.98	0.027	96880.44	0.003	yes
scalar	288.97	40	< 0.001	0.057	0.97	0.033	96895.37	0.009	yes
strict	358.18	52	< 0.001	0.055	0.96	0.037	96860.71	0.007	yes

*Notes.* χ^2^ = Chi squared; *df* = degrees of freedom; *RMSEA* = Root Mean Square Error of Approximation; *CFI* = Comparative Fit Index; *SRMR* = Standardized Root Mean Squared Residual; *BIC =* Bayesian Information Criterion; RMSEA and χ^2^ indicate Satorra–Bentler–scaled values; ∆*CFI* = Difference between Comparative Fit Indices

### Convergent and discriminant validity

**Fig. 1:**
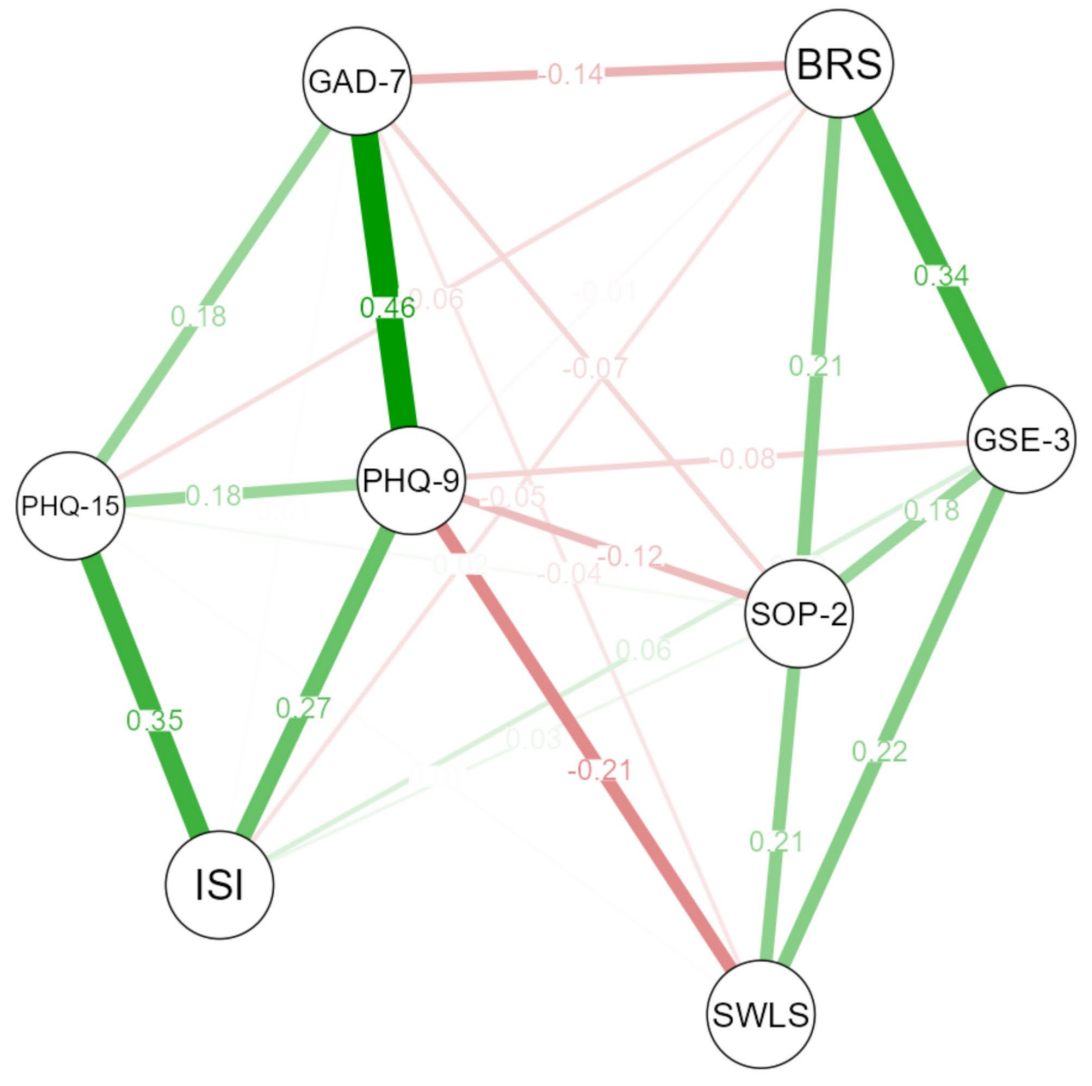
Partial correlation network constructed using EBIC–glasso depicting the association between the Brief Resilience Scale and other measures *Notes*. Each node corresponds to a measuring instrument. Green lines indicate positive association, red lines indicate negative association. *BRS* = Brief Resilience Scale; *GSE***–***3* = General Self–Efficacy Short Scale**–**3; *SOP***–***2* = Optimism–Pessimism Scale–2; *SWLS* = Satisfaction With Life Scale; *PHQ***–***9* = 9–question Patient Health Questionnaire; *ISI* = Insomnia Severity Index; *PHQ***–***15* = Patient Health Questionnaire – 15 item version; *GAD***–***7* = Generalized Anxiety Disorder 7

The results of the network analysis using the EBICglasso method revealed significant positive partial correlations between the BRS and measures of life satisfaction, social support, optimism, and self–efficacy. Suggesting that higher levels of resilience were related to increased self–efficacy, optimism, and life satisfaction. The strongest partial correlation of the BRS was observed with self–efficacy. Conversely, weak negative partial correlations were observed between the BRS and measures of depressive symptoms, anxiety, somatic symptoms, and insomnia. The network model illustrating these relationships is presented in Fig. 1.

## Discussion

This study adds to previous work on the psychometric properties of the German version of the BRS [20, 21] by examining the item characteristics, reliability, factor structure, measurement invariance, and validity in a sample of persons with mental disorders. Our findings showed that the items of the BRS exhibited good item difficulty and discriminability. When compared to the population-based sample in Chmitorz et al. [20], where mean BRS scores were generally higher, the scores in our inpatient clinical sample were lower. This finding suggests that inpatient clinical populations may exhibit lower resilience levels, which could be due to the acute stress and mental health challenges they face. These differences highlight the construct validity of the BRS, as it appears sensitive to varying levels of resilience between general and clinical populations. Reliability analyses indicated that the BRS exhibited good internal consistency, which was in line with previous studies [11, 20–22, 19]. The observed factor structure was consistent with the structure identified by Chmitorz et al. [20], confirming the presence of a two–factor structure with a general resilience factor and a method factor. The method factor accounts for variance associated with the wording of the items—whether they are positively or negatively phrased—rather than differences in resilience itself. This suggests that respondents’ answers are influenced not only by their resilience levels but also by the way the items are phrased. Recognizing the influence of item phrasing helps to ensure that the BRS accurately reflects resilience rather than response biases or tendencies towards agreeing with items regardless of content. Our findings provide evidence that the BRS shows strict measurement invariance across a diverse spectrum of diagnostic categories. These categories include a range of conditions such as affective disorders, neurotic, stress–related, somatoform disorders and behavioral syndromes. The consistent measurement invariance across these diverse diagnostic groups highlights the reliability of the BRS as a valid tool for assessing resilience as outcome, regardless of the specific mental health condition. This allows for meaningful comparisons of resilience levels between various patient groups, enabling clinicians and researchers to gain insights into the comparative strengths and vulnerabilities among these groups. The positive correlations observed between the BRS and related constructs, including well–being measures like life satisfaction, as well as resilience–promoting factors like self–efficacy and optimism, offer strong evidence that the BRS effectively captures multifaceted aspects of resilience.

The validation of the BRS in a clinical sample holds practical implications for clinicians in their daily practice. One practical implication is that the BRS provides clinicians with a standardized and economical way to assess resilience of their patients. Clinical professionals are often constrained by time limitations that hinder the collection of supplementary constructs beyond the requisite patient history questionnaires. This constraint is further compounded by the prevalent disorder–specific and disorder–oriented diagnostic focus. Its ability to capture an important resource–oriented aspect of patients’ well–being, irrespective of the disorder they might be dealing with, enhances the comprehensiveness of clinical evaluations and ensures that additional information about patients’ psychological strengths is taken into account. With its concise format and good psychometric properties, the BRS offers a useful tool that can be integrated into clinical assessments. Integrating the BRS into routine assessments could assist clinicians in gaining a more holistic insight into the strengths and vulnerabilities of individual patients. This approach has the potential to facilitate the identification of patients with potentially lower psychosocial resources, allowing for the consideration of more tailored support during therapy. Overall, the integration of the BRS into clinical practice has the potential to enhance the quality of care provided to patients and promote resilience–focused interventions.

### Strengths and limitations

The major strength of our study is the first psychometric testing of the German–language BRS in a large sample of persons with mental disorders, which underscores the practical utility of the scale for clinical applications. However, there are some limitations. First, it was conducted within a singular psychosomatic clinic, which predominantly provided behavioral therapy treatments. This may limit the diversity and representativeness of our sample, potentially affecting the generalizability of our findings to broader clinical populations or different treatment settings. Possible specific treatment approaches and patient characteristics could introduce biases that may not be reflective of the larger mental health landscape. Additionally, the sample consisted primarily of persons with depression, eating disorders, obsessive–compulsive disorder, and anxiety disorders. Therefore, the current findings may not be generalizable to people with other mental disorders, such as psychotic disorders or substance use disorders. Thus, future studies need to replicate our findings in more diverse clinical settings (e.g., out–patient settings). Second, we did not compare the BRS with other measures of resilience such as the Connor–Davidson Resilience Scale [10] or the Stressor Reactivity Score [62], which could have provided valuable insights into the scale’s concurrent validity and its distinctiveness from existing resilience measures. Third, test–retest reliability was not calculated in this study. This was because psychotherapeutic treatment was provided. This decision was made to avoid confounding measuring test–retest reliability with assessing treatment efficacy. Calculating test–retest reliability in such a scenario would have introduced the confounding factor of therapeutic effect, making it difficult to isolate the true stability of the measured variables over time. Fourthly, another limitation of this study is the timing of data collection, some of which took place during the COVID-19 pandemic. The widespread impact of the pandemic on mental health and stress levels [63] may have influenced participants’ self-reported resilience, potentially biasing the results.

## Conclusion

This study provides valuable evidence regarding the psychometric properties of the German version of the BRS in a sample of persons with mental disorders. The findings support the reliability, factorial validity, measurement invariance, and convergent validity of the BRS in assessing resilience in individuals in a clinical context. The robust psychometric properties of the BRS suggest that it could be a valuable tool for assessing resilience in clinical populations. Practitioners such as clinicians or psychotherapists can use the BRS to assess and monitor resilience as an important transdiagnostic factor in understanding and promoting psychological well–being.

## Data Availability

The datasets generated and/or analyzed during the current study are not publicly available due to confidentiality reasons as they contain clinical data but are available from the corresponding author on reasonable request.
